# Endemic Mycoses: Novel Findings for the Clinician

**DOI:** 10.3390/jof8111184

**Published:** 2022-11-10

**Authors:** Alessandro C. Pasqualotto

**Affiliations:** 1 Santa Casa de Misericordia de Porto Alegre, Porto Alegre 90035-075, Brazil; pasqualotto@santacasa.org.br; Tel.: +55-51-999951614; 2 Universidade Federal de Ciencias da Saude de Porto Alegre, Porto Alegre 90035-075, Brazil; 3 Molecular Biology Laboratory, Hospital Dom Vicente Scherer, Heliponto, Av Independencia 155, Centro, Porto Alegre 90035-075, Brazil

Endemic mycoses are difficult-to-diagnose conditions that may mimic several other diseases, particularly tuberculosis, community-acquired pneumonia, and cancer. Even though these are well-known diseases for physicians living in endemic areas, fungal diseases have now reached novel geographic areas, in a world where extensive travelling occurs. We should therefore all be aware of their existence.

The microscopic appearance of the agents of endemic mycoses is frequently suggestive, but their diagnoses require considerable expertise, and test sensitivities are variable. Culture from potentially involved sites remains the diagnostic gold-standard; however, plates need to be incubated for up to 6–8 weeks. Treatments of severe clinical forms usually rely on amphotericin B and its lipid formulations, while itraconazole is the most active oral antifungal agent. Controlled trials are rare in the field, and access to modern medicines is usually a challenge in developing countries.

With the purpose of summarizing recent findings involving the epidemiology, diagnosis and treatment of these conditions, the *Journal of Fungi* is publishing a Special Issue dedicated to endemic mycoses. A summary of the novel findings associated with some of these conditions is presented herein.

## 1. Blastomycosis

Blastomycosis is endemic in the Mississippi and Ohio River basins, in the United States. The range of disease may be expanding, with cases now commonly occurring in the state of New York, and other areas previously considered outside the traditional region of endemicity. Regarding the diagnosis of blastomycosis, an antigen detection assay is available in North America and has acceptable sensitivity (85–93%) and specificity (79–99%) [1]. Testing of the urine is preferable over other sample types.

## 2. Coccidioidomycosis

The majority of cases of coccidioidomycosis are reported in Arizona and California; the number of coccidioidomycosis cases is continuing to increase yearly in the United States. Other arid areas in Latin America are also involved. Culturing *Coccidioides* spp. from clinical samples presents biosafety concerns. Most patients are diagnosed with serology, but weeks are also required for antibodies to be produced and detected. Coccidioidal antigen testing is also available and is best performed in urine samples [2]. Antigenuria should be tested only when the diagnosis is in question.

## 3. Cryptococcosis Due to *Cryptococcus gattii*

In contrast to the opportunistic agent *C. neoformans*, which has a global distribution and is frequently isolated from bird droppings, *C. gattii* is reported in tropical and subtropical areas of the globe, and is associated with trees. Moreover, *C. gattii* may cause infection in immunocompetent individuals. Infections caused by *C. gattii* are more likely to result in mass-like lesions, either pulmonary or cerebral. *C. gattii* shows higher minimum inhibitory concentrations to several antifungals than *C. neoformans*.

## 4. Emergomycosis

Since the introduction of molecular-based identification methods of dimorphic fungi in some South African laboratories, emergomycosis has been recognized as the most frequently diagnosed endemic mycosis in South Africa [3]. To date, nearly all cases of emergomycosis have involved immunocompromised patients. *Emergomyces* spp. can cross-react with the *Histoplasma* galactomannan antigen test; therefore, urine samples can be sent for *Histoplasma* antigen testing.

## 5. Histoplasmosis

Once considered a disease restricted to the Mississippi and Ohio rivers in the United States, histoplasmosis is now a global disease, with hundreds of cases being reported in China, India, Oceania, Africa, and Europe. The highest burden of disease occurs in Latin America. As for the other agents of endemic mycoses, the growth of *Histoplasma* species in culture is slow and the detection of *Histoplasma* antigens may enable the rapid diagnosis of histoplasmosis, particularly in patients with acute or disseminated disease. Antigen testing has recently been evaluated using a lateral flow technique. Urine antigen testing is preferred. Histoplasmosis is the only endemic mycosis for which a randomized controlled trial has been conducted, revealing the superiority of liposomal amphotericin B (L-AmB) over deoxycholate amphotericin B in disseminated disease [4]. An ongoing study is evaluating the effectiveness and safety of single-dose regimens of L-AmB in AIDS patients with histoplasmosis (clinical trials identification NCT04059770), in a similar fashion to what has been performed with leishmaniasis and cryptococcosis.

## 6. Lobomycosis

Lobomycosis is a neglected fungal disease that mostly occurs in the Amazon rainforest in Brazil. Infected patients present with keloidal nodules after fungal inoculation following traumatic exposure. Even though this is not a notifiable disease, an increase in the number of cases has been observed in recent years. Diagnosis—which still relies on histopathology—is challenging, because lesions are quite often mistaken for leishmaniosis, atypical mycobacteria including leprosy, sporothrychosis, and other dermatological fungal diseases. No drug is effective for the treatment of lobomycosis, and surgical resection has usually been followed by disease recurrence. Preliminary data suggest that posaconazole might be effective in lobomycosis; however, clinical trials are lacking.

## 7. Paracoccidioidomycosis 

Outside endemic areas in Latin America, paracoccidioidomycosis is a disease carried by travelers who have lived in those areas for extended times. An expansion of endemicity has been associated with changes in agricultural practices, as well as the emergence of the newly recognized species *P. lutzii* [5]. The majority of patients with chronic disease are diagnosed using serology, or the microscopy of infected tissues or fluids. Antigen detection is not yet applicable to clinical practice, due to the lack of standardized or commercially available tests.

## 8. Sporothrychosis

Until recently, it was believed that only members of the pathogenic clade of *Sporothrix schenckii* were able to cause disease, but additional species such as *S. brasiliensis*, *S. globosa, S. mexicana*, and *S*. *pallida* have gained importance. The clinical relevance of identifying *Sporothrix* at the species level remains unclear. Serologic testing is promising, but currently limited by availability [6]. Antigen testing has the potential to be used in sporothrychosis.

## 9. Talaromycosis

Another disease which has had its name changed in mycology: penicilliosis is now talaromycosis. Initially a disease solely associated with AIDS patients, talaromycosis is now increasingly recognized in association with other immunocompromising conditions [7]. The region of endemicity is expanding, from Vietnam and southern China to northern and eastern China (Beijing and Shanghai) and in northeastern India. Mp1p antigen testing is now strongly recommended in the diagnosis of talaromycosis (superior sensitivity compared with culture: specificity > 95%) in plasma and urine specimens.

## 10. Conclusions

Most significant changes involving endemic mycoses have affected diagnostic tools. With such slow-growing fungi, this is fertile terrain for point-of-care tests to grow. Molecular assays are also in place for diagnosis, but standardization is required. With advances in fungal DNA sequencing, novel fungal species have been described, with some being of clinical or epidemiological relevance. For most of these fungi, the value of antifungal susceptibility testing is unknown, and serology remains largely unstandardized. Most pharmacological interventions in endemic mycoses are based on uncontrolled studies. Modern trials involving antifungal drugs are required in endemic mycosis. Additionally, above all, the development of drugs and diagnostic tests is only justifiable if these are made available to the countries in which these conditions are endemic, to reduce the burden of these diseases.

## Data Availability

Not applicable.
